# Renal angiomyolipoma in tuberous sclerosis complex: Case series and literature review 

**DOI:** 10.5414/CNCS110768

**Published:** 2023-03-05

**Authors:** Mansour Mbengue, Bede Bigirimana, Seynabou Diagne, Abdou Niang

**Affiliations:** Department of Nephrology, Cheikh Anta Diop University, Dakar, Senegal

**Keywords:** tuberous sclerosis complex, angiomyolipoma, angioembolization

## Abstract

Tuberous sclerosis complex (TSC) is a genetic disease characterized by the growth of numerous noncancerous tumors in many parts of the body mainly the skin, brain, kidneys. The prevalence of the disease is estimated to be 7 – 12 in 100,000. We report the cases of two black African women diagnosed with TSC at age 25 and 54. They both had renal angiomyolipoma, facial angiofibroma and diffuse hypochromic macules. The older patient remained stable for the 11 years following her diagnosis. But, in the second patient, the disease was more severe with a giant angiomyolipoma, complicated by renal intracystic hemorrhage leading to the patient’s death 1 month after diagnosis. Renal involvement can be life-threatening in patients with TSC. The risk of fatal bleeding increases with the size of the tumor. The mTOR inhibitors and angioembolization can improve the prognosis of this disease.

## Introduction 

Tuberous sclerosis complex (TSC) is a genetic disease that causes non-cancerous tumors to grow in the brain and on other vital organs such as the kidneys, heart, liver, eyes, lungs, and skin. The prevalence of the disease is estimated to be 7 – 12 in 100,000. TSC occurs in all races and ethnic groups, and in both genders [1, 2]. TSC is diagnosed with clinical and genetic tests. TSC is caused by a mutation of either of two genes, *TSC1* and *TSC2*, which code for the proteins hamartin and tuberin, respectively. These proteins act as tumor growth suppressors, agents that regulate cell proliferation and differentiation [3]. CT scans have an important role in the diagnosis by identifying the different tumor components, particularly fatty components, which is fundamental in the diagnostic criteria for angiomyolipoma [4]. Renal involvement can be life-threatening in patients with TSC due to the risk of bleeding which increases with tumor size. The mTOR inhibitors (mTORi) and angioembolization can improve the prognosis of this disease [5]. 

## Case report 1 

**Figure 1.**
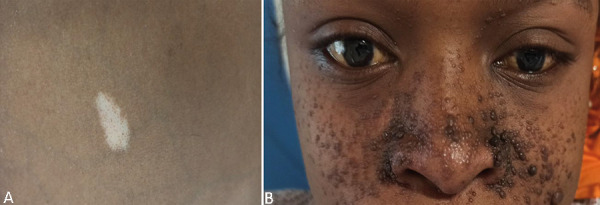
Skin examination showed (A) hypochromic macules on the trunk and (B) facial angiofibromas.

A 25-year-old black woman with no family history of genetic disease was diagnosed with multiple renal cysts. The medical background assessment showed abdominal pain since 4 years. A physical examination found blood pressure at 160/90 mmHg, pulse at 112 beats/min, respiratory rate at 22 cycles/min, and weight at 45 kg. The general condition was altered with pallor of the conjunctiva. The abdomen was distended and painful with large lumbar masses. Skin examination showed diffuse hypochromic macules on the trunk, face, and limbs and facial angiofibromas (Figure 1). The eye examination was normal. 

### Biology 

Renal failure was found with serum creatinine at 56 mg/L, blood urea at 1.3 g/L. A complete blood count showed severe anemia with a hemoglobin level of 4 g/dL. 

### Imaging 

**Figure 2.**
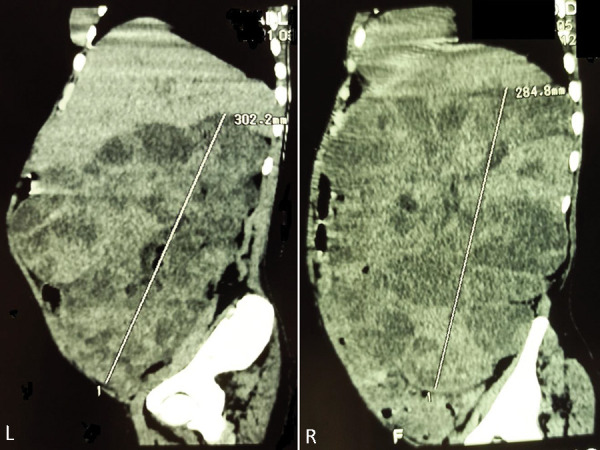
Sagittal CT scan showed two large kidneys measuring 196 × 132 × 284 mm on the right and 214 × 125 × 302 mm on the left with budding and heterogeneous masses with mixed matrix (tissue, fluid, and fat) corresponding to angiomyolipoma.

**Figure 3.**
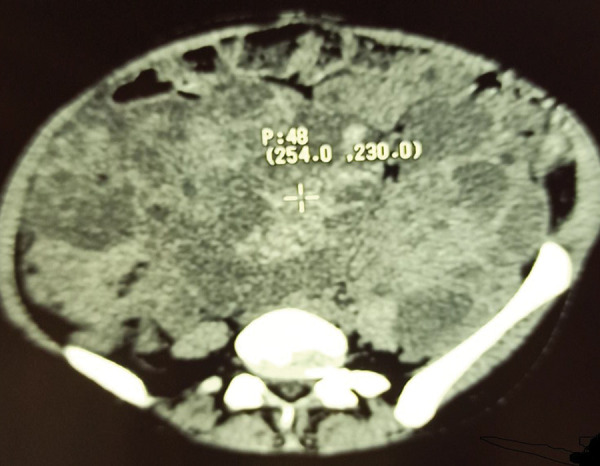
Axial CT-scan showed spontaneous intracystic hyperdensities (70 HU) corresponding to hemorrhages.

**Figure 4.**
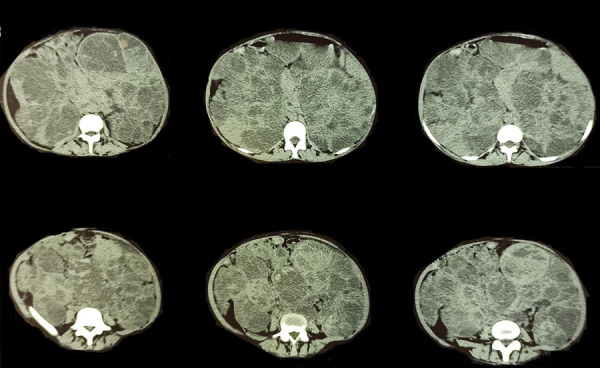
Axial CT scan showed two large kidneys with budding and heterogeneous masses with mixed matrix (tissue, fluid, and fat).

Abdominal CT scan showed two large kidneys measuring 196 × 132 × 284 mm on the right and 214 × 125 × 302 mm on the left with budding and heterogeneous masses with mixed matrix (tissue, fluid, and fat) corresponding to angiomyolipoma (Figure 2, Figure 3). There were spontaneous intracystic hyperdensities (70 HU) corresponding to hemorrhage (Figure 4). 

### Diagnostic criteria 

The diagnosis of TSC was made in this patient based on the criteria of the 2012 consensus conference on TSC. The patient met 3 major criteria. 

### Evolution 

The patient received transfusions and tranexamic acid. The high blood pressure was treated with amlodipine 10 mg/day. Angioembolization was not performed because it was not available. The outcome was marked by the patient’s death from hemorrhagic shock 1 month after diagnosis. 

## Case report 2 

A 65-year-old black woman, with no family history of genetic disease, was diagnosed with renal cysts since 8 years. Interrogation revealed abdominal pain that had been evolving for 2 years. A physical examination found blood pressure at 140/100 mmHg, pulse at 82 beats/min, respiratory rate at 18 cycles/min, and weight at 65 kg. The examination showed large lumbar masses, angiofibromas on the face, and intraoral fibromas. The rest of the examination was normal. 

### Biology 

Serum creatinine was normal at 9 mg/L, blood urea at 0.35 g/L. A complete blood count showed a hemoglobin level of 14 g/dL. 

### Imaging 

Abdominal CT scan showed two large kidneys measuring 136 × 82 × 176 mm on the right and 140 × 85 × 162 mm on the left with heterogeneous masses with mixed matrix (tissue, fluid, and fat) corresponding to angiomyolipomas. 

### Diagnostic criteria 

The diagnosis of TSC was made in this patient based on the criteria of the 2012 Consensus Conference on TSC. The patient met 2 major and 1 minor criteria. 

### Evolution 

The arterial hypertension was treated with amlodipine 10 mg/day. The outcome was favorable, marked by stabilization of renal function. 

## Discussion 

Patients with tuberous sclerosis develop tumors or abnormalities that appear at different ages and in multiple organs [6]. Renal involvement is manifested by the simultaneous presence of angiomyolipomas and cysts. Angiomyolipomas during TSC are often multiple, bilateral, and rapidly growing. These tumors are often complicated by bleeding which manifests as hematuria and exacerbation of lumbar pain resulting in Lenk’s triad (lumbar mass, pain, and hematuria). The risk of bleeding is higher with tumors larger than 4 cm, rapid tumor growth and aneurysms larger than 0.5 cm [7, 8]. This is illustrated by the two cases that we presented, because it was case 1 that had a higher tumor mass and presented with hemorrhage. These hemorrhages are favored by the appearance of the blood vessels in angiomyolipomas, which are tortuous and thick-walled with no elastic supporting tissue, which tend to form intralesional pseudoaneurysms. Histologically, angiomyolipomas are classified into two types: potentially malignant epithelioid angiomyolipomas and benign triphasic angiomyolipomas. The latter are divided into classical angiomyolipomas and low-fat angiomyolipomas. Classical angiomyolipomas are characterized by three components: vascular, fatty, and spindle cells. In contrast, low fat angiomyolipoma is defined as < 25% fat per field by light microscopy and does not contain enough fat to be detected by imaging. Kidney biopsy is contraindicated due to the high risk of bleeding [4]. CT scans have an important role in the diagnosis of angiomyolipomas. It makes it possible to confirm the diagnosis by identifying the various tumor components, in particular fatty components, which is fundamental in the diagnostic criteria for angiomyolipomas. CT scans have high accuracy and reproducibility. Its use in monitoring young patients is limited by cumulative irradiation over time. Its use is also limited in patients with glomerular filtration rate (GFR) ˂ 45 mL/min/1.73m^2^ due to the risk of contrast-induced nephropathy. Magnetic resonance imaging (MRI) thus allows a good characterization of the lesions and does not irradiate. It allows to make a good appreciation of the size of the lesions. In the updated TSC diagnostic criteria and surveillance and management recommendations from 2021, MRI of the abdomen is recommended for newly diagnosed or suspected TSC, and it is recommended to obtain MRI of the abdomen to assess for the progression of angiomyolipoma and renal cystic disease every 1 – 3 years throughout the lifetime of the patient [9]. 

Kidney biopsy should be carried out in cases in which imaging is not contributory to make the diagnosis and in cases with suspicion of malignant transformation. However, there is a high risk of bleeding. To minimize this hemorrhagic risk, transjugular biopsy is preferred to percutaneous biopsy [4]. Cutaneous involvement is frequent and is often manifested by facial angiofibromas found in ~ 90% of cases, hypochromic macules, or subungual fibromas [10]. Other organ impairments can be observed, such as:

- Brain: Cognitive disorders and convulsions.
- Heart: Cardiac rhabdomyomas can be observed leading to cardiac insufficiency.
- Eyes: Achromic retinal phacomas
- Lung: Lymphangioleiomyomatosis in adolescents.

In our cases, the diagnosis was based on the diagnostic criteria of 2012, because at the time of their consultation these criteria were current [11]. These criteria have been recently updated. The new diagnostic clinical criteria have only two changes from the previous version and now include 11 major features and 7 minor features (Table 1). The previous major clinical diagnostic criterion of “cortical dysplasias” was found to be nonspecific in practice and potentially confusing to clinicians, given that TSC is one of several causes of focal cortical dysplasias. The new criterion is “multiple cortical tubers and/or radial migration lines”, which is more specific to TSC [9]. 

Regarding the treatment of angiomyolipomas, there are two aspects to consider. First, there is the preventive treatment to reduce or avoid the increase in the size of the lesions. For this preventive component, mTORi are recommended as the first-line treatment. This preventive treatment is indicated in asymptomatic patients with growing angiomyolipoma lesions ≥ 3 cm [9, 12]. The minority of patients who develop advanced renal failure can still benefit from mTORi therapy to prevent bleeding and to possibly slow or stop decline in renal function. Early diagnosis and adequate management with mTORi would prevent complications such as hemorrhage and end-stage renal disease [9]. The second part of the treatment concerns patients who have presented a complication such as hemorrhage. In this case, arterial embolization followed by corticosteroids is indicated first [5, 9, 12, 13]. Surgical treatment is indicated in case of angioembolization failure or in case of suspected malignancy [14]. The disadvantage of surgical treatment is that it causes reduced nephron mass which may lead the patient to require renal replacement therapy in a shorter time. This risk also exists for angioembolization, but it is lower. Microwave tumor ablation, radiofrequency, and cryoablation are other possible treatments for small tumors [15]. 

## Conclusion 

TSC is a rare disease. CT scan can confirm the diagnosis by identifying the various tumor components, particularly fatty components. Kidney damage can be life-threatening in patients with TSC, because the risk of bleeding increases with the size of the tumors. mTORi and angioembolization can improve the prognosis of this disease. 

## Statement of ethics 

The present case reports adhered to the Declaration of Helsinki. Written informed consent for publication was obtained from the patients. 

## Funding 

This research received no specific grant from any funding agency in the public, commercial, or not-for-profit sectors. 

## Conflict of interest 

The authors have no conflicts of interest to declare. 

**Table 1.** Diagnostic criteria (2021) [9].

Major criteria	Minor criteria
Hypomelanotic macules (≥ 3, at least 5 mm diameter)	“Confetti” skin lesions
Angiofibromas (≥ 3) or fibrous cephalic plaque	Dental enamel pits (≥ 3)
Ungual fibromas (≥ 2)	Intraoral fibromas (≥ 2)
Shagreen patch	Retinal achromic patch
Multiple retinal hamartomas	Multiple renal cysts
Multiple cortical tubers and/or radial migration lines	Non renal hamartomas
Subependymal nodules (≥ 2)	Sclerotic bone lesions
Subependymal giant cell astrocytoma	
Cardiac rhabdomyoma	
Lymphangioleiomyomatosis*	
Angiomyolipomas (≥ 2)*	

Definite TSC: 2 major features or 1 major feature with 2 minor features. Possible TSC: either 1 major feature or ≥ 2 minor features. Genetic diagnosis: A pathogenic variant in TSC1 or TSC2 is diagnostic for TSC (most TSC-causing variants are sequence variants that clearly prevent TSC1 or TSC2 protein production. Some variants compatible with protein production (e.g., some missense changes) are well established as disease-causing; other variant types should be considered with caution). *A combination of the 2 major clinical features lymphangioleiomyomatosis and angiomyolipomas without other features does not meet the criteria for a definite diagnosis.
